# Supplementary material for: Optimization of the Fermentation Conditions of Metarhizium robertsii and Its Biological Control of Wolfberry Root Rot Disease
Source: Microorganisms. 2023 Sep 23;11(10):2380. doi: 10.3390/microorganisms11102380 (PMC10609576; doi:10.3390/microorganisms11102380)
Supplement: Supplementary file 1 [file microorganisms-11-02380-s001.zip › microorganisms-2622701-supplementary.pdf]

Table S1

Table S1. Codes and levels of factors for response surface methodology experiments

Level	Factor		
	**A**	**B**	**C**
	**pH**	**Rotational speed (rpm)**	**Temperature (℃)**
-1	6	180	28
0	7	190	29
1	8	200	30

Table S2

Table S2. Experimental design and results of strain culture conditions

Number	Factor			Inhibition rate (%)
	A<br>pH	B<br>Rotational speed (rpm)	C<br>Temperature (℃)	
1	-1	-1	0	43.79
2	1	-1	0	33.48
3	-1	1	0	38.46
4	1	1	0	40.01
5	-1	0	-1	41.83
6	1	0	-1	32.97
7	-1	0	1	45.78
8	1	0	1	38.70
9	0	-1	-1	32.80
10	0	1	-1	30.18
11	0	-1	1	39.82
12	0	1	1	40.42
13	0	0	0	50.08
14	0	0	0	53.24
15	0	0	0	51.62
16	0	0	0	51.40
17	0	0	0	52.19

Table S3

Table S3. Regression analysis of experimental results based on Box-Behnken design

Source	Sum of squares	DF	Mean square	F-value	P-value
Model	886.29	9	98.48	34.03	＜0.0001**
A-pH	76.26	1	76.26	26.36	0.0013*
B-speed	0.084	1	0.084	0.029	0.8695
C-temperature	90.72	1	90.72	31.35	0.0008*
AB	35.16	1	35.16	12.15	0.0102*
AC	0.79	1	0.79	0.27	0.6170
BC	2.59	1	2.59	0.90	0.3754
A^2	80.70	1	80.70	27.89	0.0011*
B^2	296.60	1	296.60	102.51	＜0.0001**
C^2	237.35	1	237.35	82.03	＜0.0001**
Residual	20.25	7	2.89		
Lack of Fit	14.92	3	4.97	3.73	0.1179
Pure Error	5.33	4	1.33		
Total	906.55	16			
R^2	0.9777				

Note:* indicates significant difference at $P<0.05$ and ** indicates extremely significant difference at $P<0.01$.
